# Silver Nanoparticles Mediated by *Costus afer* Leaf Extract: Synthesis, Antibacterial, Antioxidant and Electrochemical Properties

**DOI:** 10.3390/molecules22050701

**Published:** 2017-04-29

**Authors:** Elias E. Elemike, Omolola E. Fayemi, Anthony C. Ekennia, Damian C. Onwudiwe, Eno E. Ebenso

**Affiliations:** 1 Material Science Innovation and Modelling (MaSIM) Research Focus Area, Faculty of Agriculture, Science and Technology, North-West University, Mafikeng Campus, Private Bag X2046, Mmabatho 2735, South Africa; chemphilips@yahoo.com (E.E.E.); omololaesther12@gmail.com (O.E.F.); eno.ebenso@nwu.ac.za (E.E.E.); 2 Department of Chemistry, School of Mathematics and Physical Sciences, Faculty of Agriculture, Science and Technology, North-West University, Mafikeng Campus, Private Bag X2046, Mmabatho 2735, South Africa; 3 Department of Chemistry, College of Science, Federal University of Petroleum Resources, P.M.B 1221 Effurun, Delta State, Nigeria; 4 Department of Chemistry, Federal University Ndufu-Alike Ikwo (FUNAI), P.M.B 1010 Abakiliki, Ebonyi State, Nigeria; chemisttony@gmail.com

**Keywords:** silver nanoparticles, *Costus afer*, electrochemical, antioxidant, antibacterial

## Abstract

Synthesis of metallic and semiconductor nanoparticles through physical and chemical routes has been extensively reported. However, green synthesized metal nanoparticles are currently in the limelight due to the simplicity, cost-effectiveness and eco-friendliness of their synthesis. This study explored the use of aqueous leaf extract of *Costus afer* in the synthesis of silver nanoparticles (CA-AgNPs). The optical and structural properties of the resulting silver nanoparticles were studied using UV-visible spectroscopy, scanning electron microscopy (SEM), transmission electron microscopy (TEM) and Fourier transform infra–red spectrophotometer (FTIR). TEM images of the silver nanoparticles confirmed the existence of monodispersed spherical nanoparticles with a mean size of 20 nm. The FTIR spectra affirmed the presence of phytochemicals from the *Costus afer* leaf extract on the surface of the silver nanoparticles. The electrochemical characterization of a CA-AgNPs/multiwalled carbon nanotubes (MWCNT)-modified electrode was carried out to confirm the charge transfer properties of the nanocomposites. The comparative study showed that the CA-AgNPs/MWCNT-modified electrode demonstrated faster charge transport behaviour. The anodic current density of the electrodes in Fe(CN)_6_]^4−^/[Fe(CN)_6_]^3−^ redox probe follows the order: GCE/CA-Ag/MWCNT (550 mA/cm^2^) > GCE/MWCNT (270 mA/cm^2^) > GCE (80 mA/cm^2^) > GCE/CA-Ag (7.93 mA/cm^2^). The silver nanoparticles were evaluated for their antibacterial properties against Gram negative (*Escherichia coli, Klebsiella pneumonia, Pseudomonas aeruginosa*) and Gram positive (*Bacillus subtilis* and *Staphylococcus aureus*) pathogens. The nanoparticles exhibited better inhibition of the bacterial strains compared to the precursors (leaf extract of *Costus afer* and silver nitrate). Furthermore, the ability of the nanoparticles to scavenge DPPH radicals at different concentrations was studied using the DPPH radical scavenging assay and compared to that of the leaf extract and ascorbic acid. The nanoparticles were better DPPH scavengers compared to the leaf extract and their antioxidant properties compared favorably the antioxidant results of ascorbic acid. The green approach to nanoparticles synthesis carried out in this research work is simple, non-polluting, inexpensive and non-hazardous.

## 1. Introduction

Nanoparticles with controlled size, shape and structure are important building blocks for many optical, magnetic, electronic, and biomedical applications [1]. The organization and patterning of silver NPs (AgNPs) into one and two-dimensional functional templates have recently attracted much attention due to their potential advantages as photosensitive components of optical devices, electrocatalysts, chemical sensors and antibacterial agents [1,2,3,4]. The synthesis of silver nanoparticles can be achieved by using various methods which involve chemical reduction [5], photochemical reduction [6], electrochemical reduction [7], and heat vaporization [8]. These processes all involve different toxic chemicals as reducing agents or stabilization agents for the silver ion. In order to circumvent the complication of toxicity in the synthesis of silver nanoparticles due to their potential biological applications, plants or plant extracts have been established to have a leading role in the AgNP bio synthesis process [9]. Phytosynthesis (the use of plant extracts in synthesis of nanoparticles) of metal nanoparticles incorporates the use of weed, seeds, gums, fruits, roots, bark, sap and flowers [9]. In using this approach for synthesis, the properties of the nanoparticles could be tuned by changing the ratio of the precursor salts to the substrates; and the method is simple, practicable, sustainable and cost effective. Currently several AgNPs have been synthesized using different plant extracts as potential reducing agents [9,10].

Biosynthesized silver nanoparticles have been reported to exhibit a number of biological properties that includes antimicrobial [10,11,12], antioxidant [11,12,13,14], would healing [9] and anticancer effects [11,15]. In addition, silver catalysts have been successfully used in heterogeneous catalysis at an industrial scale for the oxidation of methanol to formaldehyde and ethylene to ethylene oxide [16]. Similarly, Ag-doped semiconductor nanoparticles have many applications in photocatalysis (i.e., degradation of organic pollutants, hydrogen production, CO_2_ photoreduction, disinfection), in order to improve the photo-conversion yield and allow the extension of the light absorption of wide band gap semiconductors to the visible light [17,18]. Recent reports suggest that the removal of organic dyes using Ag nanoparticles is a better choice than the common dye removal techniques like redox treatment, electrocoagulation, carbon sorption, and UV photodegradation [2].

The adsorption of chemical species on the surface of nanotubes is an area of immense interest due to its relevance for molecular electronics and because it is a necessary prerequisite for any surface-mediated chemical process [19]. Carbon nanotues (CNT) possessing high thermal capacity and ability to promote electron transfer reactions present an enormous potential as inorganic templates [20]. This is explored in the preparation of AgNPs/CNT hybrid materials, which integrate the unique physical and chemical properties of each component [21] Silver nanoparticles (AgNPs) attached onto multi-walled carbon nanotubes (MWCNTs) have attracted significant attention due to their unique properties [22,23]. The modification of electrodes used in electrochemical experiments using various multifunctional materials in order to confer novel electrochemical properties to the electrode has become a common practice in electrochemistry studies [24,25,26]. Multi-walled carbon nanotubes (MWCNTs) have been used to modify the electrode surfaces in order to increase the sensitivity towards the electrochemical determination of some compounds and enzymes [25,26]. However, literature on the infusion of green synthesized silver nanoparticles on carbon nanotubes is still scarce. Similarly, there are not many reports on the electrochemistry of these hybrid nanocomposites from plant-mediated nanoparticles [27,28,29]. Hence, in this work we utilized the active components of the leaf extracts of *Costus afer* for nanoparticle synthesis and investigated in a comparative study the electrochemical properties of a bare glassy carbon electrode and an electrode modified with CA-AgNPs, MWCNT and CA-AgNPs/MWCNT nanomatrices as potential electrochemical sensors for biological and environmental molecules.

*Costus afer* belongs to the family *Costaceae*, a monocot and a relatively tall, herbaceous, unbranched tropical plant with creeping rhizomes. It is commonly found in moist or shady and river banks forest of West and Tropical African countries including Senegal, South Africa, Guinea, Nigeria, Ghana and Cameroon [30]. Almost all parts of *Costus afer* have been extensively used in traditional medicine as a remedy for cough, rheumatic pains, sleepiness and as a cardiotonic [31]. The phytochemical composition of extracts of different parts of the plant has been established and reported [31,32]. The phytochemical analysis of the leaves and stem aqueous extracts showed some phytocomponents such as alkaloids, saponins, tannins, flavonoids and phenols. Alkaloids are associated with anti-inflammatory, anti-microbial effects as well as anti-hypertensive agent, and the healing effect has earlier been attributed to the presence of tannins in in the plant. The roots of *Costus afer* have been reported to contain oxalic acid, lanosterol, trigogenin, a new diosgenin, stigmasterol, sitosterol, costugenin and a new steroid saponinaferoside A [32]. Leaves of another species, *Costus pictus*, have been reported to contain flavonoids such as kaempferol, 3,4-di *O*-Me-quercetin and phenolic acids such as 2,5-dihydroxybenzoic acid, *O*-coumaric acid, *p*-hydroxybenzoic acid that possess good antioxidant, anti-inflammatory, hypoglycemic and renal functions [14,33]. The vast medicinal applications of extracts of *Costus afer* prompted the antimicrobial and antioxidant evaluation of silver nanoparticles synthesized by the aqueous leaf extract of this plant material. The result was compared with that obtained from bare aqueous leaf extract of *Costus afer* and standard drugs.

## 2. Results and Discussion

### 2.1. UV-Visible Spectroscopy

**Figure 1:**
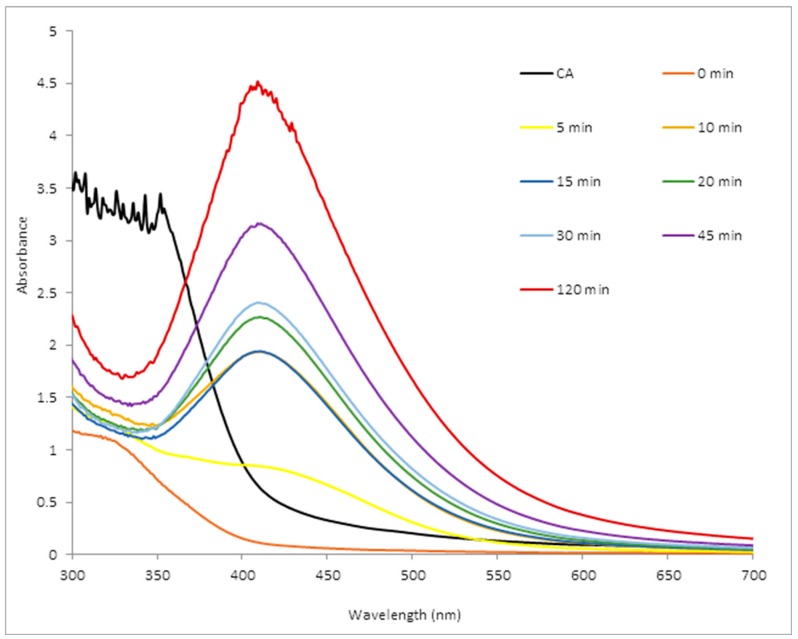
UV-vis spectra showing the surface plasmon resonance exhibited by the nanoparticles at different time intervals.

UV-visible spectroscopy is a valuable technique used to detect the characteristic surface plasmon resonance (SPR) pattern of metal nanoparticles. Metal nanoparticles exhibit SPR phenomena when metal electrons in the conduction band collectively oscillate in resonance with certain wavelengths of incident light. The synthesis was monitored in a time dependent manner using UV-visible spectroscopy as shown in Figure 1.

The UV-visible spectra showed an early appearance of a surface plasmon resonance peak (SPR) with high intensity after 10 min. However, as the reaction time increased, the sharpness of the SPR peak increased too. This is often a factor of the size, shape dispersion; nature of biomolecules and surrounding media characteristics of the phytochemical reducing agent, as different extracts give varying SPR results [34,35,36]. The range of the wavelengths throughout the incubation time was within 405–411 nm, with continuous increase in intensity which reflects the presence of a considerable amount of monodispersed and stable AgNPs. AgNPs absorb radiation intensely within the observed wavelengths. The exact mechanism for the extracellular biosynthesis of metal nanoparticles is not well understood, but studies are in progress to unravel the mechanism of the crystal growth and how to control the size, as well as how to develop particles of higher monodispersity [37].

### 2.2. FTIR Analysis

**Figure 2:**
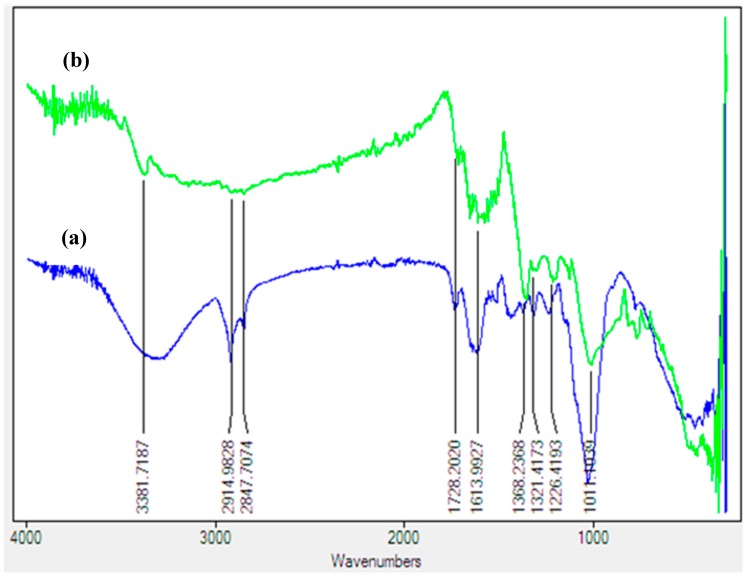
The FTIR spectra of the (**a**) Costus afer leaf and (**b**) biosynthesized AgNPs.

FTIR spectroscopy was used to identify the possible biomolecules present in *Costus afer* leaf powder which are responsible for reducing the Ag^+^ to Ag^0^, capping and stabilizing the silver nanoparticles. The FTIR spectra recorded for the leaf and CA-AgNPs are presented in Figure 2.

The FTIR spectrum of leaf powder showed peaks at 3307, 1625, 1375, and 1029 cm^−1^ which are similar to peaks observed in CA-AgNPs. Only a slight variation at the ligating sites manifests that biomolecule adsorption on the silver occurred. The vibrational peak around 3307 cm^−1^ is assigned to ν(O–H) stretching that could possibly emanate from carbohydrates or phenolics. The peaks found around 2917 and 2849 cm^−1^ are ascribed to the –C-H stretch of the alkyl group, and the peak around 1625 cm^−1^ is due to the enolic β-diketones or -C=O stretch of carboxylic acids, while the corresponding -C-O stretch is observed around 1375 and 1029 cm^−1^. The assignment of peaks is similar to other literature reports [38,39]. The appearance of these peaks suggested the presence of phytochemicals such as flavonoids, alkaloids, phenolics, organic acids of the *Costus afer* leaf in the CA-AgNPS, which are responsible for stabilizing as well as the capping effects on the nanoparticles. The biomolecules may have interacted with the Ag ions through their oxygen donor atoms and are adsorbed on the surface of the metal ions which is manifested by a decrease in the peak intensities of bands observed in the AgNPs [34].

### 2.3. Microscopy/Compositional Analysis

**Figure 3:**
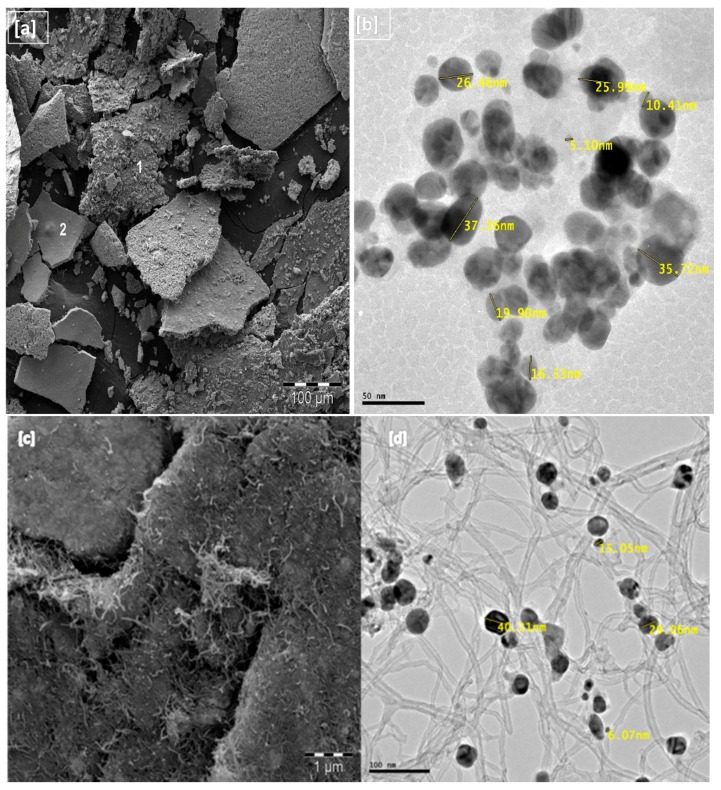
(**a**) SEM and (**b**) TEM images of the as-synthesized *Costus afer* mediated silver nanoparticles (**c**) SEM and (**d**) TEM images of the CA-AgNPs/MWCNT.

Scanning electron microscopy (SEM) and transmission electron microscopy (TEM) were used to study the surface morphology, shape and size of the nanoparticles and the nanocomposite. The images are presented in Figure 3a–d. The SEM image showed high density silver nanoparticles with surface morphology which reveals encapsulation of the biomass in the silver. Bio-capped molecules help to prevent agglomeration of nanoparticles and also enhance antimicrobial activity [40,41]. Semi-quantitative estimation of the nanoparticles composition was performed by EDS and the respective spots of the phytocapped CA-AgNPs are represented by labels 1 and 2 in Figure 3a. Quantitative analysis provides the weight % of all the elements present in the biosynthesized AgNPs as: C (8.51%), Mg (0.16%), Cl (3.77%), Ag (60.33%), O (27.24%) and for the nanocomposites: C 61.95%, O 9.52%, Mg 0.22%, Cl 1.73%, K 1.07%, Ag 25.51%. The presence of O, Cl, C and Mg peaks along with Ag signals, suggest that the AgNPs are bonded to phytoconstituents of the leaf extract. The Ag detection limit is much higher than for all other trace elements present in the AgNPs while in the CA-AgNPs/MWCNT nanocomposite, a higher percentage of carbon was recorded. Hence, Ag spots are prevalent in the elemental mapping. Similar works have reported the synthesis of AgNPs using extracts of *A. farnesiana*, *Dracoce phalummoldavica*, pine, persimmon, magnolia, ginkgo, platanus, and guava extracts which showed the presence of C, O and other minor elements with Ag having higher percentage composition [42,43,44,45,46]. The TEM analysis showed well stabilized particles with spherical shape and negligible aggregation. As shown in Figure 3b, the size of the AgNPs is about 5–38 nm range. Figure 3d shows the intercalation of the AgNPs with the multiwalled carbon nanotube signifying the well dispersed nature of the composite and the interactive mode leading to enhanced electrochemical detection.

### 2.4. Thermal and EDX Analysis

**Figure 4:**
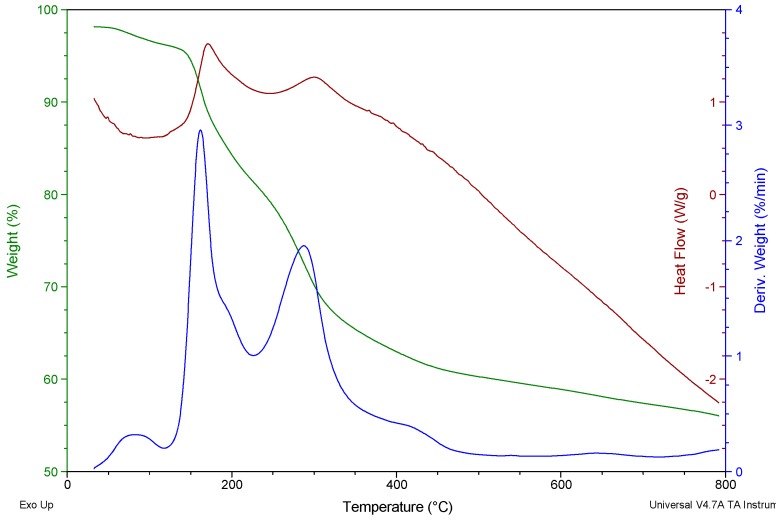
TG/DTG and DSC curves of the CA-AgNPs obtained in nitrogen atmosphere.

Figure 4 shows the TGA/DSC/DTG graph of CA-AgNPs. The graph presents a three step decomposition process. The first step of the trace, which occurred in the 50–115 °C range, was due to the evaporation of water moisture and residual solvent molecules adsorbed on the surface of AgNPs. The second step weight loss between 116–225 °C, and the third step between 228–480 °C were consequences of the surface desorption of bio-organic compounds present in the nanoparticle powder. These suggest that there were more than one type of organic molecules acting as capping materials. Silver NPs stabilized by plant leaf extract are expected to be made up of different molecules responsible for the reduction of metal ion and also stabilizing the particles in the solution [47]. Overall, the TGA results show a loss of about 40.5% weight up to 480 °C. According to the DSC curve, the elimination of the water molecules was an endothermic curve with peak temperature around 95 °C, while the desorptions of the biomolecules were exothermic reactions with peak temperatures around 169 and 300 °C. This result suggests that the phytochemicals which are responsible for the reduction of Ag^+^ to Ag^0^ (nanoparticle) are strongly coordinated to the silver and that they are relatively stable compounds. The residue remaining after heating is a pure silver microstructure. The EDX analysis carried out on the nanoparticles before thermal analysis confirmed the presence of C, O, Cl, Mg and Ca which are due to the plant material. The composition of the plant may differ based on the environment and geographical regions [48]. The presence of C and O supports the FTIR results which suggested carbohydrates or phenolics as components of the plant extracts and attachment of the plant extract to the silver was via oxygen.

### 2.5. Electrochemical Studies of the AgNPs Mediated Nanocomposites

**Figure 5:**
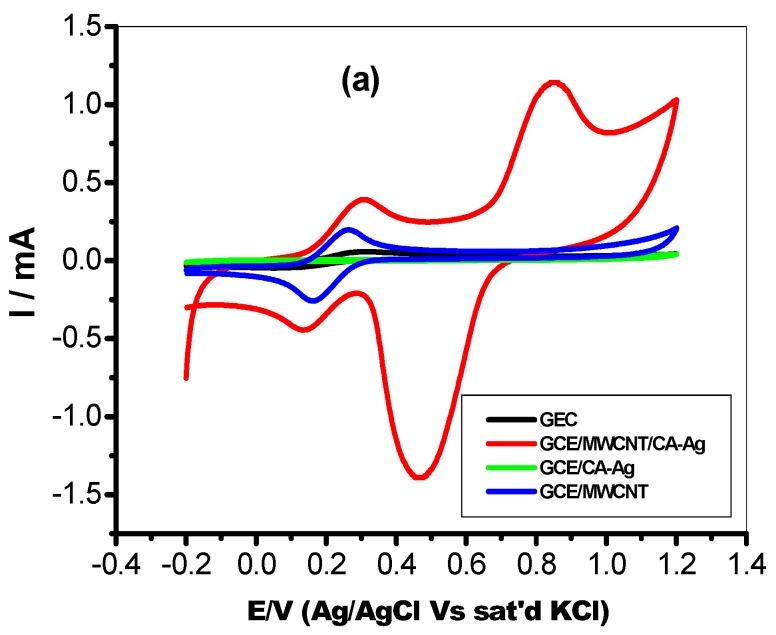
Cyclic Voltammograms of GCE/CA-Ag (green), bare GCE (black), GCE/MWCNT (blue), and GCE/CA-Ag/MWCNT (red) in 5mM Fe(CN)_6_]^4−^/[Fe(CN)_6_]^3−^ solution prepared in 0.1 M PBS at scan rate 25 mVs^−1^.

A comparative study on the charge transfer properties of the bare and modified glassy carbon electrodes was performed with cyclic voltammetry experiments. The CV were performed in 5 mM Fe(CN)_6_]^4−^/[Fe(CN)_6_]^3−^ in 0.1 M PBS (scan rate, 25 mVs^−1^) and the result is presented in Figure 5. All the electrodes, including the bare GCE, showed redox peaks labelled AA’. This redox peak was attributed to the Fe(CN)_6_]^4−^/[Fe(CN)_6_]^3−^ redox process. The peak current responses of the glassy carbon-modified electrodes followed the order: CA-AgNPs/MWCNT (391 µA) > MWCNT (192 µA) > GCE (57.1 µA) > CA-AgNPs (5.63 µA). There were no other observable peak at bare GCE, CA-AgNPs and MWCNT, while on the CA-AgNPs/MWCNT modified electrodes, the second pair of redox peaks BB’ (Figure 4) in the regions of 0.86 mV and 0.47 mV were attributed to the silver nanoparticles redox process. This comparative study shows that the CA-AgNPs/MWCNT-modified glassy carbon electrode demonstrated faster charge transport behaviour which is evidenced in the observed anodic current response. The anodic current density of each modified electrode in Fe(CN)_6_]^4−^/[Fe(CN)_6_]^3−^ redox probe follows the order: CA-AgNPs/MWCNT (550 mA/cm^2^) > MWCNT (270 mA/cm^2^) > GCE (80 mA/cm^2^) > CA-AgNPs (7.93 mA/cm^2^). Therefore the glassy carbon electrode modified with CA-AgNPs/MWCNT remains the best electrode owing to the electron transport properties with current density higher than that of other modified electrodes. The study further shows the distribution of CA-AgNPs in the carbon nanotubes as shown in Figure 3d.

The synergy between MWCNT and CA-AgNPs nanocomposite in enhancing the electron transport process obtained at the GCE modified electrodes is well pronounced. The conductive nature of multiwalled carbon nanotube thus improves the properties of green plant extract-mediated silver nanoparticles and the ionic interaction between the CA-AgNPs nanocomposite and MWCNTs which results in significant electron transport at the GCE/CA-AgNPs/MWCNT-modified electrodes. Similarly, the large surface area created by the porous MWCNT on the electrode for free flow of electrolytes and charges between the base electrode and the electroactive species at the electrode surface is also another important factor for the better performance of GCE/CA-AgNPs/MWCNT electrodes. The peak-to-peak potential separation (ΔEp) for the CA-AgNPs/MWCNT electrodes is ≥ 100 mV, which is greater than the theoretical 59.8 mV expected for a fast one-electron transport. Also, the ratios of the anodic to the cathodic peak current response (Ipa/Ipc) for CA-AgNPs/MWCNT electrode are approximately unity, indicative of a reversible electrochemical process.

**Figure 6:**
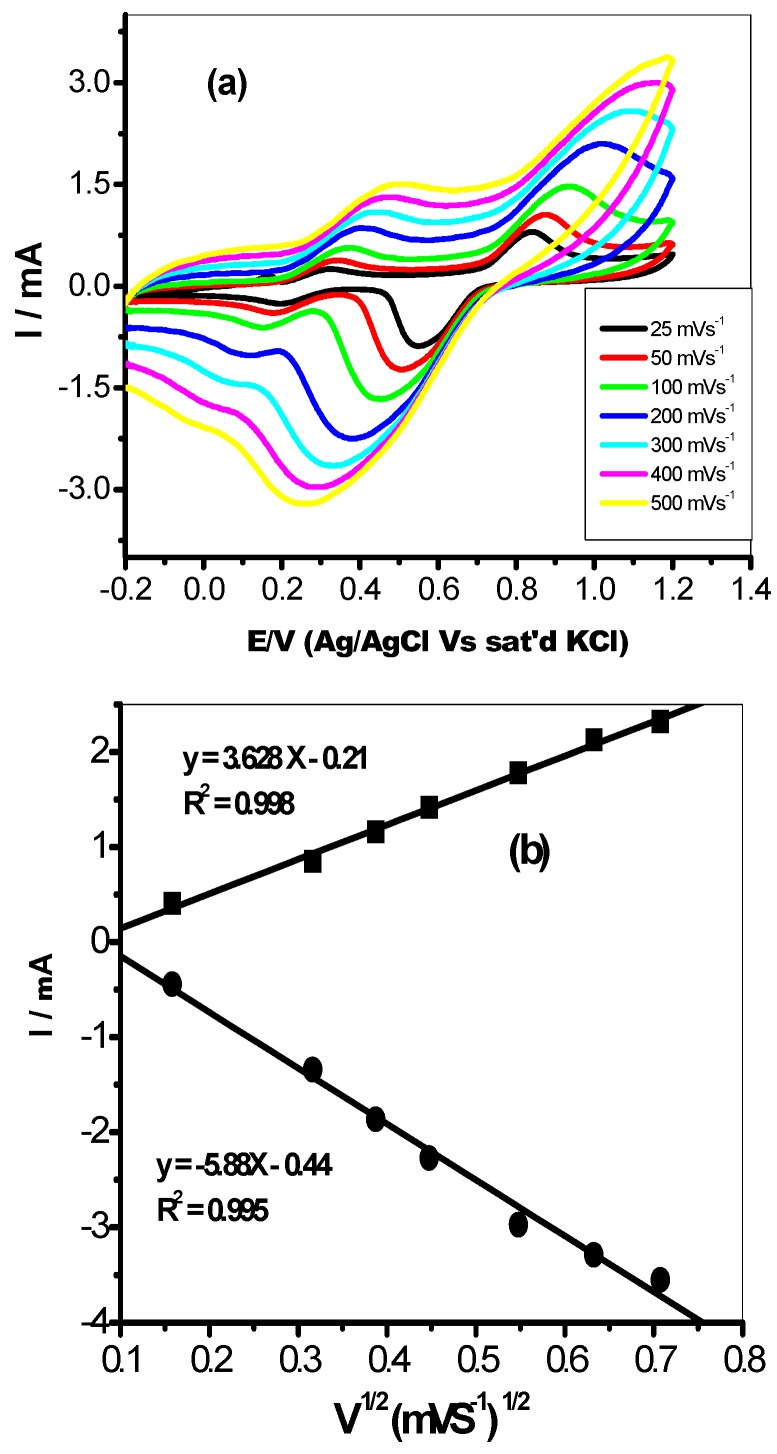
Cyclic voltammograms obtained for (**a**) GCE/CA-AgNPs/MWCNT in FECN solution prepared in 0.1 M PBS (scan rate: 25–500 mVs^−1^; inner to outer); (**b**) Linear plots of I_pa_ vs. V^1/2^ and I_pc_ vs. V^1/2^ for GCE/CA-AgNPs/MWCNT in 5 mM Fe(CN)_6_]^4−^/[Fe(CN)_6_]^3−^ solution prepared in 0.1 M PBS.

Furthermore, the effect of changes in scan rate from 25–500 mVs^−1^ on the anodic current (Ipa) and cathodic current (Ipc) at the modified CA-AgNPs/MWCNT electrode was studied for the same probe using cyclic voltammetry experiments. Both the anodic current (Ipa) and cathodic current (Ipc) increased with increase in scan rates as shown in Figure 6a. The plot of anodic and cathodic peak current (Ipa) varies linearly with the increase in scan rates (25–500 mVs^−1^, Figure 6b), with regression values of R^2^ = 0.998 and 0.995 for anodic and cathodic plots respectively, confirming a diffusion controlled process.

In addition, the peak potential moved to more positive values as the scan rate increased. This is shown in the linear correlation of the graph of peak potential (Ep) against the logarithm of scan rate (log v). The slope of Ep vs. log v for the modified electrode CA-AgNPs/MWCNT was 0.10 V. From the slope obtained, the Tafel value (b) was estimated by using the expression presented in Equation (1) [49].

(1) $$E_{P}=(\frac{b}{2})\;\log\;(v/m{\mathrm{Vs}}^{-1})+\mathrm{constant}$$

From the equation, the value of b for this electrode was calculated as 200 mVdec^−1^. This value is higher than the theoretical 118 mVdec^−1^ for a one-electron process involved in the rate-determining step. Therefore the high Tafel values suggest adsorption of reactants or intermediates on the electrode surfaces and/or reactions occurring within a porous electrode structure [50]. This process at the modified CA-AgNPs/MWCNT electrode can be linked with the porous CNT layer [49].

### 2.6. Electrochemical Impedance Spectroscopic (EIS) Studies

**Figure 7:**
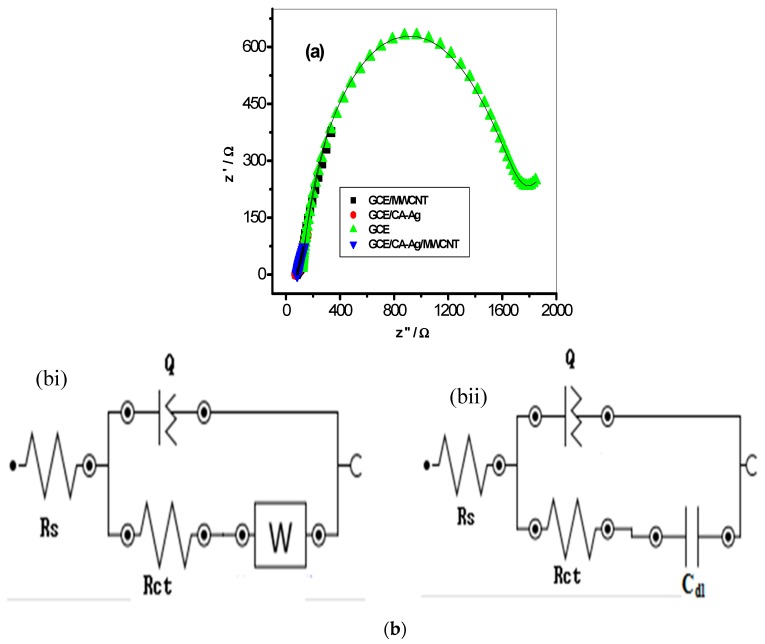
Typical Nyquist plots (**a**) obtained for the electrodes in 5 mM Fe(CN)_6_]^4−^/[Fe(CN)_6_]^3−^ solution prepared in 0.1 M PBS (pH 7) at a fixed potential of 0.2 V (vs. Ag|AgCl, saturated KCl) (**b**(**i**,**ii**)) represent the circuits used in the fitting of the EIS data.

Further investigation on the electron transport phenomenon at the electrode-electrolyte interface was carried out by using electrochemical impedance spectroscopic (EIS) techniques [51,52] in 5 mM [Fe(CN)_6_]^4−^/[Fe(CN)_6_]^3−^ solution at a fixed potential of 0.2 V. The Nyquist plots obtained from the impedance experiment (at fixed potential of 0.2 V vs. Ag/AgCl, sat’d KCl) between 10 KHz and 0.1 Hz are presented in Figure 7a for the glassy carbon modified electrode CA-AgNPs/MWCNT, CA-AgNPs, MWCNT and bare GCE, respectively. The fitting of the impedance data was done by using circuit model in the Nova 1.6 Software presented in Figure 7b(i,ii). In this circuit model, Rs is the solution resistance, C_dl_ represents the double layer capacitance, Q or CPE is the constant phase element, and Rct is the charge transfer resistance. The circuit RQ ([RC]) Figure 7b(i) was used for CA-AgNPs/MWCNT, MWCNT and CA-AgNPs, while R(Q[RW]) Figure 7b(ii) was used for the bare GCE electrode. The impedance parameters obtained from the fitting of the raw impedance spectra with the circuits are presented in Table 1. The electron transfer resistance Rct value recorded for the modified CA-AgNPs/MWCNT electrode had better electron transport with lower Rct values compared with the bare and CA-AgNps electrodes, as shown in Table 1, and these results agreed with that from CV where the conductive properties of the MWCNT influence the increase in current response recorded at the modified CA-AgNPs/MWCNT electrode.

**Figure 8:**
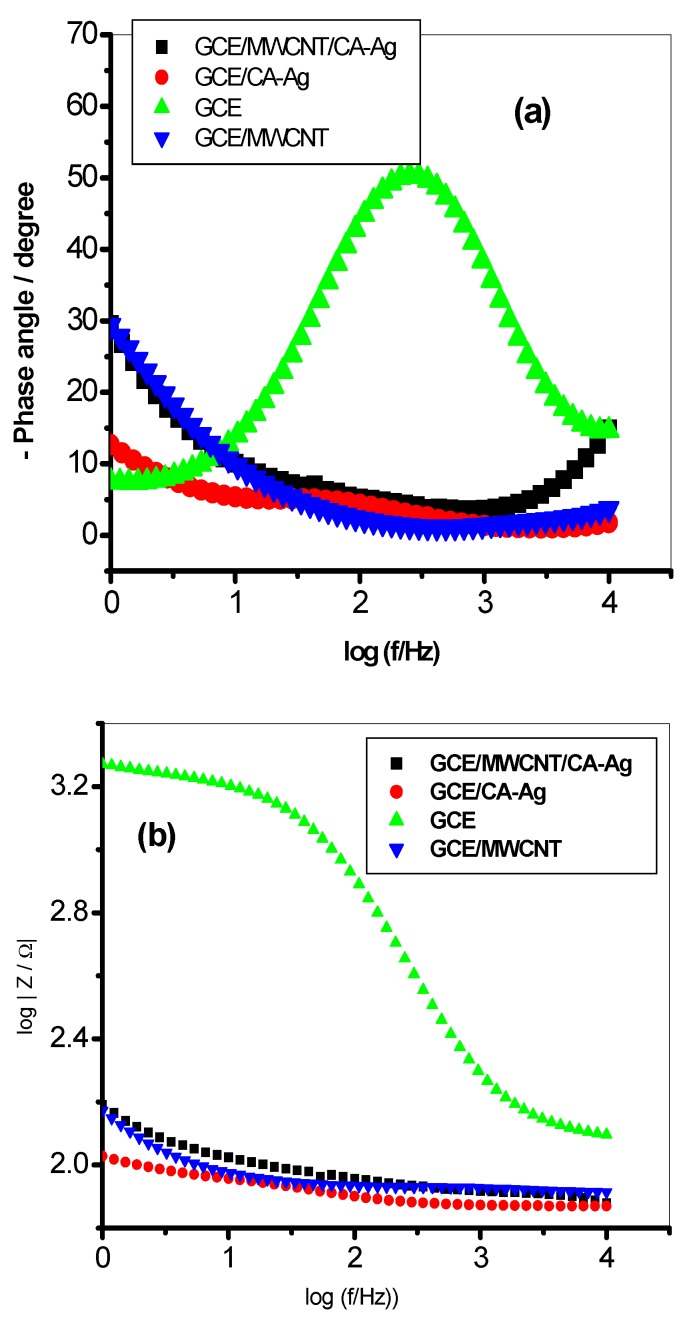
Bode plots obtained for some of the electrodes, showing (**a**) the plots of phase angle/deg. vs. log (f/Hz)) and (**b**) the plot of log |Z/Ω| vs. log (f/Hz).

The Bodes plots (Figure 8a,b) show the capacitive (charge storage) or resistance to charge transfer behaviour of some of the modified electrodes. The CA-AgNPs/MWCNT, CA-Ag and MWCNT-modified electrodes have lower phase angles, 4.3°, 4.6° and 1.3° respectively compared with the bare GCE (50.4°). The results also agreed with the Rct values which imply a faster charge transfer process at the CA-AgNPs/MWCNT-modified glassy carbon electrode. Thus, the lower the phase angle, the lower the capacitive behaviour and the higher the conductive or charge transfer properties of the sensor [53].

The charge transfer resistance (Rct) values calculated after fitting the EIS data to the Randle's equivalent circuit in Table 1 suggests the suitability of GCE/CA-AgNPs/MWCNT as a good conducting platform for electrocatalysis. This behaviour may be due to the synergy between the highly conducting silver nanoparticles and the porous conducting MWCNT with large surface area, thus facilitating more charge flow (at lower charge transfer resistance) between the electrolyte and the GC electrode. The result was further supported by the higher current response recorded at GCE/CA-AgNPs/MWCNT electrode from the CV experiment. The electrochemical study therefore proves that functionalization of CA-AgNPs with conducting material such as MWCNT to improve its electrochemical properties established the fact that modified CA-AgNPs/MWCNT electrode can be used as potential electrochemical sensors for the detection of biological molecules.

### 2.7. Antibacterial Studies

**Figure 9:**
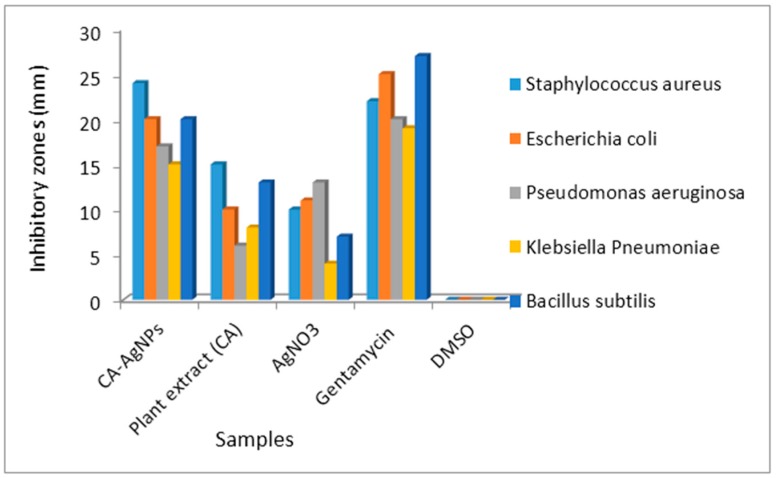
Histogram representation of the antibacterial results.

One of the main objectives of bioinorganic and medicinal chemists is to develop substances with high therapeutic values [54]. The rising prevalence of multi-drug resistant bacterial infections in the past few decades has become a serious healthcare problem. A growing number of immuno-compromised patients (with immune responses attenuated by the administration of immunosuppressive drugs) as a result of cancer chemotherapy, organ transplantation and HIV infection are the major factors contributing to this increase [55]. Hence there will always be a vital need to discover new chemotherapeutic agents to avert the emergence of bacterial resistance and ideally shorten the duration of therapy [56,57]. Several green-mediated silver nanoparticles have been reported in literature as exhibiting antibacterial activity [58,59,60,61]. Most often, the nanoparticles showed greater antibacterial activity compared to their precursor plant extracts. Antibacterial evaluation of CA-AgNPs was carried out and the results are presented in Figure 9 and Table 2.

The antibacterial results of the nanomaterial were compared to the metal free *Costus afer* extract and gentamycin (as a control drug). At a concentration of 100 μg/mL in DMSO (as a diluent), the CA-AgNPs were active to both the Gram negative (*Escherichia coli, Klebsiella pneumonia, Pseudomonas aeruginosa*) and Gram positive (*Bacillus subtilis* and *Staphylococcus aureus*) pathogens used for the antibacterial screening. The nanoparticles showed greater antibacterial activity compared to the plant extract of *Costus afer* and also compared favourably with the observed antibacterial activity of Gentamycin. The enhanced antibacterial activity of the CA-AgNPs could be attributed to their liposolubility. This is because, according to Overtone’s concept of cell permeability, the lipid membrane that surrounds the microbial cell favours the passage of only the lipid-soluble substances. Hence lipophilicity is a major property for antibiotics. In addition, Tweedy’s chelation theory proposed that on chelation of metal ions by ligands (constituent of plant extract) causes the polarity of the metal ion to be reduced to a greater extent due to the orbital overlap (between the metal and ligand orbitals) and partial sharing of the positive charge of the metal ion with donor groups of the ligands. This increases the delocalization of π-electrons over the whole chelate ring and enhances the lipophilicity of the metal complexes. The increased lipophilicity enhances the penetration of the metal complexes through lipid membranes thereby blocking the metal binding sites in the enzymes of microorganisms. In addition, it disturbs the respiration process of the cell and blocks the synthesis of the proteins, thereby, restricts further growth of the organism [62]. The CA-AgNPs and the plant extract were most active against *Staphylococcus aureus* with inhibitory zones of 24 and 15 mm, respectively. Furthermore, against *Staphylococcus aureus*, the nanoparticles were more active than Gentamycin and also exhibited 85% of the antibacterial activity of Gentamycin against *Escherichia coli*. The results clearly showed that the antibacterial activity of the nanoparticles cannot be attributed to the nature of the plant extract and AgNO_3_ individually as the CA-AgNPs showed greater activity compared to them. In addition, the antibacterial activity of the silver nanoparticles could also be attributed to the presence of some phytochemical components of the plant extract as shown by the FTIR spectra. Alkaloids are known to have antimicrobial, antifungal and anti-inflammatory effect [63], flavonoids and tannin are used for the treatment of diabetes [64] and antioxidant properties, while phenols are also known to exhibit antioxidant properties [65].

The antibacterial potentials of the CA-AgNPs were further investigated at different concentrations in order to determine the minimum inhibitory concentration of the nanoparticles for the different bacteria strains. The lowest concentration in which the nanoparticles remained active against a bacteria strain is its minimum inhibitory concentration (MIC). From Table 3, the nanoparticles were most active against *Staphylococcus aureus* and *Escherichia coli* compared to the other bacterial strains. They gave the lowest MIC for the two bacterial strains. From the obtained results, the nanoparticles were not active against *Klebsiella pneumoniae* beyond the concentration of 40 μg/mL.

### 2.8. Statistical Analysis of the Antibacterial Results

The statistical analysis of the antibacterial data was carried out using one way ANOVA to determine whether there were statistically significant differences between the groups mean values obtained from the antimicrobial studies. The significance values were found to be below 0.05; therefore, there are statistical significant differences in the different group means. Hence a post hoc test was carried out using Tukey HSD test in order to ascertain which of the specific groups differed. The results of the post hoc test were presented in Table 2 as superscript letters. Mean values in the same column with similar superscripted alphabets were not significantly different, while those with different alphabets were significantly different. From the Table, we could infer that the silver nanoparticles presented the best antimicrobial results and their mean values were significantly different from those of AgNO_3_ and plant extract (CA) against the different microbes.

### 2.9. Antioxidant Studies

Oxidative metabolism is essential for the survival of cells, although side effects of oxidative changes could lead to the production of free radicals and other reactive oxygen species. When an excess of free radicals is formed, they can overwhelm protective enzymes such as superoxide dismutase, catalase and peroxidase and cause destructive and lethal cellular effects (e.g., apoptosis) by oxidizing membrane lipids, cellular proteins, DNA and enzymes, thus shutting down cellular respiration. Oxidative stress has been implicated in illness like cancer, heart diseases, rheumatoid arthritis, etc. [66]. An antioxidant may be defined as “any substance that when present at low concentrations, compared with those of the oxidizable substrate, significantly delays or inhibits oxidation of that substrate”. DPPH radical scavenging assay is an efficient method that has been used to investigate the antioxidant capacity of many antioxidants. DPPH is a stable paramagnetic free radical, which can accept an electron or hydrogen radical in turn gets converted into a stable diamagnetic molecule [67].

**Figure 10:**
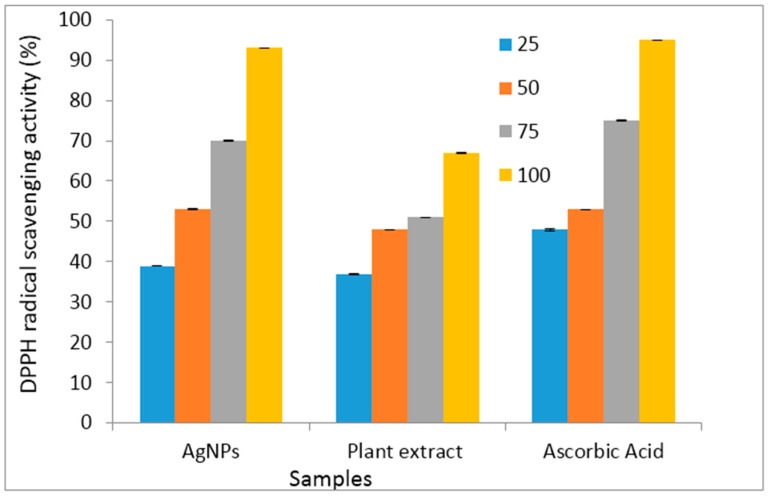
Histogram presentation of DPPH radical scavenging results.

The antioxidant potentials of CA-AgNPs were investigated and compared to that of the plant extracts of *Costus afer* and ascorbic acid (an antioxidant used as a control). The results are presented as a histogram in Figure 10. The CA-AgNPs exhibited good DPPH radical scavenging capacity. The results also reflected the dose dependence of the antioxidant properties of the samples as there was increase in percentage DPPH radical scavenging abilities as their concentrations (25–100 μg/mL) increased. The silver nanoparticles were better DPPH radical scavengers compared to the plant extract. The enhanced antioxidant capacity of CA-AgNPs can be attributed to the presence of some on the phytochemicals of *Costus afer* in the nanoparticles as capping agents. Some of which are flavonoids with several hydroxyl groups. The presence of these phytochemicals and silver ions could result to antioxidant activities proceeding through hydrogen atom transfer (HAT) and single electron transfer (SET) mechanisms simultaneously [68]. The antioxidant capacity of the CA-AgNPs was comparable to that of ascorbic acid.

### 2.10. Statistical Analysis of the Antioxidant Results

Table 4 shows the statistical analysis of the antioxidant results with the output of the ANOVA analysis and Tukey HSD test results. The significance values obtained from one way ANOVA were below 0.05; therefore, there were significant differences in the different means. The Tukey HSD test gave an insight to where those significant differences were found within different groups. Mean values within a sample group with similar superscripted letters were not significantly different, while those with different letters are significantly different. The antioxidant results obtained showed that there were significant difference between the activity of the nanoparticles, leaf extract and ascorbic acid. The nanoparticles showed greater antioxidant property than the leaf extract and compared favourably to ascorbic acid.

## 3. Materials and Methods

### 3.1. Materials

Silver nitrate (AgNO_3_) was purchased from Merck SA (Modderfontein, South Africa), and the pristine multiwalled carbon nanotubes (MWCNTs) were obtained from Sigma Aldrich (Johannesburg, South Africa). A potential glassy carbon electrode (GCE, 3 mm diameter), Ag/AgCl, sat’d KCl reference electrode, and a platinum disk counter electrode (99.999%) were purchased from CH Instrument Inc. 0.1 M Phosphate buffer solution of pH 7 (sodium hydrogen phosphate dihydrate (NaH_2_PO_4_·2H_2_O) and disodium hydrogen phosphate dihydrate (Na_2_HPO_4_·2H_2_O)) was used. Ultrapure water of 18.2 MΩcm resistivity was obtained from a Milli-Q Water System (Millipore Corp., Bedford, MA, USA) and was used throughout for the preparation of electrochemical solutions. All solutions were prepared using double distilled deionized water and purged with pure nitrogen to eliminate oxygen and any form of oxidation during experiment. The chemicals were analytical grades and were used as received.

### 3.2. Preparation of Plant Material

*Fresh leaves of Costus afer* were collected from Warri, in Delta State, Nigeria and washed several times with distilled water to remove dust particles. The leaves were identified by a taxonomist from the Department of Botany, Delta State University. The leaves were air dried and ground to powder using mortar and pestle. About 2 g of the powdered plant material was weighed and macerated with approximately 150 mL de-ionized water and heated at 90 °C for 1 h. The extract was separated by filtration and used for the nanoparticle synthesis.

### 3.3. Synthesis of Silver Nanoparticles (CA-AgNPs)

In a typical synthetic procedure, 80 mL of aqueous extract of *Costus afer* leaves was added to 400 mL of 1 × 10^−3^ M silver nitrate solution. The resulting solution was heated at 90 °C with continuous stirring for 120 min. The formation of AgNPs was accompanied by change in colour of the solution from colourless to dark brown within 120 min of reaction time. The appearance of brownish colour is a clear indication of the formation of AgNPs which was ascribed to surface plasmon resonance. The progress of the nanoparticles synthesis was monitored by UV-vis spectroscopy. The AgNPs suspension thus obtained was separated from water through repeated centrifugation at 4000 rpm for 1 h and oven-dried at 50 °C.

### 3.4. Characterization of the CA-AgNPs

The size and morphology of AgNPs and CA-AgNPs/MWCNTs was determined by using a model JEOL2100 TEM instrument (München, Germany) fitted with a LaB 6 electron gun at 5 kV, and the images were captured using an Ultrascan digital camera (Gatan, München, Germany). Samples were prepared after sonication for 1 h by placing a drop of fresh suspension on the TEM copper grids, followed by solvent drying and evaporation. The surface morphology of the AgNPs was observed using a Quanta FEG 250 Environmental Scanning electron microscope (ESEM, Hillsboro, OR, USA) under an acceleration voltage of 30 kV. Powdered samples were placed on the sample stumps and a thin gold layer was deposited on the samples to improve the electrical conductivity for better imaging. Simultaneously, the energy dispersive spectrum (EDS) was also recorded. FTIR spectroscopy measurements were carried out to identify the functional groups which are bound distinctively on the AgNPs surface and involved in the synthesis of AgNPs. Samples for the FTIR analysis were recorded using an alpha-P FT-IR spectrophotometer (Bruker, Bryanston, Sandton, Gauteng, South Africa.) in the wavenumber range 400–4000 cm^−1^. Thermal analysis was performed using a TG/DSC SDT600 thermogravimetric analyzer (TA instruments, New Castle, DE, USA) under nitrogen flow.

### 3.5. Electrochemical Experiment

The electrochemical experiment was performed on a AUTOLAB Potentiostat PGSTAT 302 (Eco Chemie, Utrecht, The Netherlands) driven by the GPES software version 4.9 in an electrochemical workstation consisting of three-electrode system, a glassy carbon electrode (GCE) of diameter 0.3 mm as the working electrode, a silver-silver chloride electrode (SCE) as the reference electrode, and a platinum (Pt) wire as the counter electrode. All the experiments were carried out at room temperature and the working solutions properly de-aerated to avoid contamination. 5 mM of Fe(CN)_6_]^4−^/[Fe(CN)_6_]^3−^ prepared with 0.1 M PBS at pH 7 was used as probe. Data fitting was performed using Nova 1.6 Software (Eco Chemie).

### 3.6. Preparation of Modified Electrodes

The GCE was first polished using 0.05 µm alumina slurry. The electrodes was then sonicated in ethanol and double distilled water for 3 min and dried at room temperature. The modified electrodes of GCE/CA-AgNPs, GCE/MWCNT and GCE/CA-AgNPs/MWCNT were prepared by casting 20 mL of CA-AgNPs, MWCNT and CA-AgNPs/MWCNT suspension on the bare GCE surface and dried in an oven at 50 °C for 5 min for the solvent to evaporate.

### 3.7. Antibacterial Analysis

Antimicrobial screening was carried out using disc diffusion method [69] Petri discs were prepared with 20 mL of sterile Mueller–Hinton agar (MHA). The test cultures were swabbed on the top of the solidified media and allowed to dry for 15 min. Specific amount (25 μL from 100 μg/mL) of CA-AgNPs, Plant extract (CA) and AgNO_3_ were introduced individually into different discs. The loaded discs were placed on the surface of the medium and left for 30 min at room temperature. The plates were incubated for 24 h at 37 °C for bacteria growth. Zones of inhibition were recorded in millimeters and the experiment was repeated twice. The bacteria strains were Gram negative (*Escherichia coli, Klebsiella pneumonia, Pseudomonas aeruginosa)* and Gram positive (*Bacillus subtilis* and *Staphylococcus aureus*) pathogens. Gentamycin was used as the positive control drug and DMSO was used as the negative control for antibacterial screening. Experimental results were given as mean ± S.D. of the two parallel measurements. Analysis of variance was performed by ANOVA procedures. Significant differences between means were determined by Tukey’s HSD tests. *p* values of < 0.05 were regarded as significant.

### 3.8. Minimum Inhibitory Concentration (MIC) Studies for CA-AgNPs

Minimum inhibitory concentration studies for CA-AgNPs was performed according to the standard methods [70] Different concentrations (10–50 μg/mL) of the nanoparticles in DMSO were prepared from the stock solution. They were added to each medium in different plates. An inoculum of 100 μL from each plate was inoculated. The MIC of the CA-AgNPs for bacteria strains at different concentrations was determined as the lowest concentration of the compound inhibiting the visual growth of the test cultures on the agar plate.

### 3.9. Antioxidant Studies: 2,2-Diphenyl-1-Picrylhydrazyl (DPPH) Free-Radical Scavenging Assay

DPPH is a stable free radical that has been widely used as a tool to estimate the free-radical scavenging activity of antioxidants [71,72,73]. The reduction capacity of the DPPH radical was determined by the decrease in absorbance induced by antioxidants following the method of Brands-Williams et al. [74] with few modifications. The reaction system consisted of 0.1 mL CA-AgNPs, plant extract (CA) and standard (ascorbic acid) diluted to different concentrations (25, 50, 75 and 100 μg/mL) and 2.9 mL of 0.025 g/L DPPH in DMSO. The mixture was shaken vigorously and left to stand at room temperature in the dark for 30 min. The absorbance was measured at 515 nm against a blank. The ability to scavenge the DPPH radical was calculated using the following formula:

(2) $$\mathrm{DPPH scavenging effect (\%)}=\frac{A_{0}-A_{1}}{A_{0}}\times 100$$

where *A*_0_ is the absorbance of the control at 30 min and *A*_1_, the absorbance of the CA-AgNPs and plant extract (CA) at 30 min.

### 3.10. Statistical Analysis

Statistical analysis was performed with one-way analysis of variance (ANOVA). For statistical studies the SPSS 17.0 software (IBM cooperation, North Castle, NY, USA) was used. Three replications for each of the experiments and assays were conducted (*n* = 3). A mean of the three values was reported in each case. The values are expressed as Mean ± Standard deviation. A post Hoc test was performed by the use of Tukey’s HSD test.

## 4. Conclusions

Monodispersed, stable, electrochemically and biological active AgNPs have been prepared using *Costus afer* extract as a bioreductant and stabilizing agent in a simple, low cost and environment-friendly biological approach. The plant extract demonstrated the ability to actively cap and produce nanoparticles of controlled size and morphology. TEM results revealed spherically shaped CA-AgNPs in a highly dispersed manner at low concentration of plant extract and perfect encapsulation in the nanocomposites. The cyclic voltammetry comparative study at the glassy carbon modified electrodes for doped and undoped nanocomposite of CA-AgNPs with MWCNT revealed that the presence of MWCNT improved the electrochemical properties of the silver nanoparticles prepared from *Costus afer* extract. This is evidenced by the improved current response of the glassy carbon electrode and the value of the electron transfer resistance. The electrochemical studies suggest that the CA-AgNPs-MWCNT-modified electrode may have application as a sensor for the assay of both biological and environmental samples. The CA-AgNPs showed greater antibacterial and antioxidant activity compared to the precursor plant extract. The nanoparticles were more active than Gentamycin against *Staphylococcus aureus*, and compared favourably against other bacterial strains. This green AgNPs synthesis method may find use in applications such as biomedical, electrochemical and environmental as they do not require the use of any toxic reagent and therefore should be further explored.

## Figures and Tables

**Table 1:** Impedance data obtained for the bare GCE and the modified electrodes in 5 mM Fe(CN)_6_]^4−^/[Fe(CN)_6_]^3−^ solution at 0.2 V (vs. Ag|AgCl saturated KCl). All values were obtained from the fitted impedance spectra. The values in parentheses are percent errors of data fitting.

**Electrodes**	**Electrochemical Impedance Spectroscopy Data**			
	**R_s_ (Ω)**	**Q (×10^6^ nF)**	**R_ct_ (Ω)**	**W × 10^2^**
GCE	116.10 (1.04)	4.53 (4.05)	1572.00 (1.14)	0.14 (10.97)
**Electrodes**	**Electrochemical Impedance Spectroscopy Data**			
	**R_s_ (Ω)**	**Q (×10^6^ nF)**	**R_ct_ (Ω)**	**C_dl_ (uF)**
GCE/MWCNT	82.30 (0.59)	0.36 (3.33)	15.00 (27.05)	302.10 (23.24)
GCE/CA-AgNPs	72.20 (0.50)	0.66 (2.52)	76.50 (4.36)	399.00 (4.65)
GCE/CA-AgNPs/MWCNT	76.10 (2.37)	0.21 (5.46)	74.70 (11.74)	100.30 (9.79)

**Table 2:** Descriptive table for antibacterial studies.

Microbial Strains	MIC	Mean	Std. Deviation	Sig
Bacterial Strains	Samples			
*Staphylococcus aureus*	CA-AgNPs	23.00 ^a^	1.414	0
	Plant extract (CA)	13.00 ^b^	2.828	
	AgNO_3_	8.00 ^b,c^	2.828	
	Gentamycin	21.50 ^a,d^	0.707	
	DMSO	00.00 ^e^	0	
*Escherichia coli*	CA-AgNPs	20.50 ^a^	0.707	0
	Plant extract (CA)	10.50 ^b^	0.707	
	AgNO_3_	12.00 ^b,c^	1.414	
	Gentamycin	24.00 ^a,d^	1.414	
	DMSO	00.00 ^e^	0	
*Pseudomonas aeruginosa*	CA-AgNPs	17.50 ^a^	0.707	0
	Plant extract (CA)	7.50 ^b^	2.121	
	AgNO_3_	14.00 ^a,c^	1.414	
	Gentamycin	20.00 ^a,d^	0	
	DMSO	00.00 ^e^	0	
*Klebsiella pneumoniae*	CA-AgNPs	14.00a	1.414	0.001
	Plant extract (CA)	11.00 ^a,b^	4.243	
	AgNO_3_	4.00 ^b,c^	0	

Values (in mm) represent the mean of double replications and their standard deviation; Gentamycin; Standard antibacterial drug used as positive control for antibacterial studies, Negative control DMSO and R = resistant. Post Hoc analysis (Tukey HSD test): Mean values in the same column with similar super scripted alphabets were not significantly different, while those with different alphabets are significantly different.

**Table 3:** MIC values of silver nanoparticles (CA-AgNPs).

*Staphylococcus aureus*	10 μg/mL
*Bacillus subtilis*	25 μg/mL
*Pseudomonas aeruginosa*	25 μg/mL
*Escherichia coli*	10 μg/mL
*Klebsiella pneumoniae*	40 μg/mL

**Table 4:** Antioxidant results.

Concentration (μg/mL)	CA-AgNPs	Sig	Plant Extract (CA)	Sig	Ascorbic Acid	Sig
25	39 ± 0.107 ^a^	0.000	37 ± 0.154 ^a^	0.027	48 ± 0.257 ^a^	0.001
50	53 ± 0.134 ^a^		48 ± 0.017 ^a,b^		53 ± 0.035 ^a,b^	
75	70 ± 0.108 ^b^		51 ± 0.058 ^a,b,c^		75 ± 0.045 ^c^	
100	93 ± 0.068 ^c^		67 ± 0.172 ^b,c^		95 ± 0.003 ^d^	

Mean values are the percentage DPPH radical scavenging ability of the samples at different concentrations. Post Hoc analysis (Tukey HSD test): Mean values in the same column with similar super scripted alphabets were not significantly different, while those with different alphabets are significantly different.

## References

[1] Zhihong N., Alla P., Eugenia K. (2010). Properties and emerging applications of self-assembled structures made from inorganic nanoparticles. Nat. Nanotechnol..

[2] Vanaja M., Paulkumar K., Baburaja M., Rajeshkumar S., Gnanajobitha G., Malarkodi C., Sivakavinesan M., Annadurai G. (2014). Degradation of methylene blue using biologically synthesized silver nanoparticles. Bioinorg. Chem. Appl..

[3] Kaushik R., Sarkar C.K., Ghosh C.K. (2015). Photocatalytic activity of biogenic silver nanoparticles synthesized using yeast (*Saccharomyces cerevisiae*) extract. Appl. Nanosci..

[4] Salata O.V. (2004). Applications of nanoparticles in biology and medicine. J. Nanobiotechnol..

[5] Tan Y., Wang Y., Jiang L., Zhu D. (2002). Thiosalicylic acid-functionalized silver nanoparticles synthesized in one-phase system. J. Colloid Interface Sci..

[6] Mallick K., Witcomb M.J., Scurrell M.S. (2005). Self-assembly of silver nanoparticles in a polymer solvent: Formation of a nanochain through nanoscale soldering. Mater. Chem. Phys..

[7] Liu Y.C., Lin L.H. (2004). New pathway for the synthesis of ultrafine silver nanoparticles from bulk silver substrates in aqueous solutions by sonoelectrochemical methods. Electrochem. Commun..

[8] Smetana A.B., Klabunde K.J., Sorensen C.M. (2005). Synthesis of spherical silver nanoparticles by digestive ripening, stabilization with various agents, and their 3-D and 2-D superlattice formation. J. Colloid Interface Sci..

[9] Yugal K., Mohanta S., Sujogya K.P., Rasu J., Nanaocha S., Akshaya K.B., Tapan K.M. (2017). Antimicrobial, antioxidant and cytotoxic activity of silver nanoparticles synthesized by leaf extract of *Erythrina suberosa* (Roxb.). Front Mol. Biosci..

[10] Ahmed S., Ahmad M., Swami B.L., Ikram S. (2016). A review on plants extract mediated synthesis of silver nanoparticles for antimicrobial applications: A green expertise. J. Adv. Res..

[11] Venkatesan J., Kim S.K., Shim M.S. (2016). Antimicrobial, antioxidant, and anticancer activities of biosynthesized silver nanoparticles using marine algae ecklonia cava. Nanomaterials.

[12] Kokila T., Ramesh P.S., Geetha D. (2016). Biosynthesis of AgNPs using Carica Papaya peel extract and evaluation of its antioxidant and antimicrobial activities. Ecotoxicol. Environ. Saf..

[13] Kumar B., Kumari S., Luis C. (2016). Biosynthesis of silver nanoparticles using lavender leaf and their applications for catalytic, sensing, and antioxidant activities. Nanotechnol. Rev..

[14] Nakkala J.R., Bhagat E., Suchiang K., Sadras S.R. (2015). Comparative study of antioxidant and catalytic activity of silver and gold nanoparticles synthesized from *Costus pictus* leaf extract. J. Mater. Sci. Technol..

[15] Luo Z., Zheng K., Xie J. (2014). Engineering ultrasmall water-soluble gold and silver nanoclusters for biomedical applications. Chem. Commun..

[16] Albiter E., Valenzuela M.A., Alfaro S., Valverde-Aguilar G., Martínez-Pallares F.M. (2015). Photocatalytic deposition of Ag nanoparticles on TiO_2_: Metal precursor effect on the structural and photoactivity properties. J. Saudi Chem. Soc..

[17] Ansari S.A., Khan M.M., Ansari M.O., Lee J., Cho M.H. (2013). Biogenic synthesis, photocatalytic, and photoelectrochemical performance of Ag-ZnO nanocomposite. J. Phys. Chem..

[18] Khan M.M., Ansari S.A., Amal M.I., Lee J., Cho M.H. (2013). Highly visible light active Ag@TiO_2_ nanocomposites synthesized using an electrochemically active biofilm: A novel biogenic approach. Nanoscale.

[19] Liu G., Lin Y. (2006). Amperometric glucose biosensor based on self-assembling glucose oxidase on carbon nanotubes. Electrochem. Commun..

[20] Wang J., Li M., Shi Z.J., Li N., Gu Z. (2002). Direct electrochemistry of cytochrome c at a glassy carbon electrode modified with single-wall carbon nanotubes. Anal. Chem..

[21] Moghaddam M.J., Taylor S., Gao M., Huang S., Dai L., McCall M.J. (2004). Highly efficient binding of DNA on the sidewalls and tips of carbon nanotubes using photochemistry. Nano Lett..

[22] Guo D.J., Li H.L. (2005). Highly dispersed Ag nanoparticles on functional MWNT surfaces for methanol oxidation in alkaline solution. Carbon.

[23] Chin K.C., Gohel A., Chen W.Z., Elim H.I., Ji W., Chong G.L., Sow C.H., Wee A.T.S. (2005). Gold and silver coated carbon nanotubes: An improved broad-band optical limiter. Chem. Phys. Lett..

[24] Mohan S., Okomu F., Oluwafemi O.S., Matoetoe M., Arotiba O. (2016). Electrochemical behaviour of silver nanoparticle-MWCNTs hybrid nanostructures synthesized via a simple method. Int. J. Electrochem. Sci..

[25] Umasankar Y., Unnikrishnan B., Chen S., Ting T. (2012). Effective determination of acetaminophen present in pharmaceutical drug using functionalized multi-walled carbon nanotube film. Int. J. Electrochem. Sci..

[26] Silveira C.M., Pimpão M., Pedroso H.A., Rodrigues P.R.S., Moura J.J.G., Pereira M.F.R., Almeida M.G. (2013). Probing the surface chemistry of different oxidized MWCNT for the improved electrical wiring of cytochrome *c* nitrite reductase. Electrochem. Commun..

[27] Leopold N., Lendl B. (2003). A new method for fast preparation of highly surface-enhanced Raman scattering (sers) active silver colloids at room temperature by reduction of silver nitrate with hydroxylamine hydrochloride. J. Phys. Chem..

[28] Merga G., Wilson R., Lynn G., Milosavljevic B.H., Meisel D. (2007). Redox catalysis on “naked” silver nanoparticles. J. Phys. Chem..

[29] Evanoff D.D., Chumanov G. (2004). Size-controlled synthesis of nanoparticles. 1. “silver-only” aqueous suspensions via hydrogen reduction. J. Phys. Chem..

[30] Edeoga H.O., Okoli B.E. (2000). Chromosome numbers of *Costus lucanusianus* (Costaceae) in Nigeria. Folia Geobot..

[31] Momoh S., Yusuf O.W., Adamu M.M., Agwu C.O.C., Atanu F.O. (2011). Evaluation of the phytochemical composition and hypoglycaemic activity of methanolic leaves extract of *Costus afer* in albino rats. Br. J. Pharm. Res..

[32] Ukpabi C.F., Agbafor K.N., Ndukwe K.O., Agwu K., Nwachukwu S.N. (2012). Phytochemical components of *Costus afer* extracts and its alleviation of carbon tetrachloride induced hepatic oxidative stress and toxicity. Int. J. Mod. Bot..

[33] Ajithadas A., Ramraj N., Venkatachalam K., Pandi B., Shanmuganathan J., Kannappan V. (2014). Insulin plant (*Costus pictus*) leaves: Pharmacognostical standardization and phytochemical evaluation. Am. J. Pharm. Health Res..

[34] Khan Z.U.H., Khan A., Shah A., Wan P., Chen Y., Khan G.M., Khan A.U., Tahir K., Muhammad N., Khan H.U. (2016). Enhanced photocatalytic and electrocatalytic applications of green synthesized silver nanoparticles. J. Mol. Liq..

[35] Dwivedi A.D., Gopal K. (2010). Biosynthesis of silver and gold nanoparticles using *Chenopodium album* leaf extract. Colloids Surf. A.

[36] Christopher J.G., Saswati B., Ezilrani P. (2015). Optimization of parameters for biosynthesis of silver nanoparticles using leaf extract of *Aegle marmelos*. Braz. Arch. Biol. Technol..

[37] Okafor F., Janen A., Kukhtareva T., Edwards V., Curley M. (2013). Green synthesis of silver nanoparticles, their characterization, application and antibacterial activity. Int. J. Environ. Res. Public Health.

[38] Tripathy A., Raichur A.M., Chandrasekaran N., Prathna T.C., Mukherjee A. (2010). Process variables in biomimetic synthesis of silver nanoparticles by aqueous extract of *Azadirachta indica* (Neem) leaves. J. Nanopart. Res..

[39] Verma A., Mehata M.S. (2016). Controllable synthesis of silver nanoparticles using Neem leaves and their antimicrobial activity. J. Rad. Res. Appl. Sci..

[40] Gnanadhas D.P., Ben Thomas M., Thomas R., Raichur A.M., Chakravortty D. (2013). Interaction of silver nanoparticles with serum proteins affects their antimicrobial activity in vivo. Antimicrob. Agents Chemother..

[41] Abdel-Mohsen A.M., Hrdina R., Burgert L., Abdel-Rahman R.M., Hasova M., Smejkalova D., Kolar M., Pekar M., Aly A.S. (2013). Antibacterial activity and cell viability of hyaluronan fiber with silver nanoparticles. Carbohydr. Polym..

[42] Deshpande L.M., Chopade B.A. (1994). Plasmid mediated silver resistance in *Acinetobacter baumannii*. Biometals.

[43] Yallappa S., Manjanna J., Dhananjaya B.L. (2015). Phytosynthesis of stable Au, Ag and Au-Ag alloy nanoparticles using *J. Sambac* leaves extract, and their enhanced antimicrobial activity in presence of organic antimicrobials. Spectrochim. Acta.

[44] Leu J.G., Chen S.A., Chen H.M., Wu W.M., Hung C.F., Yao Y.D., Tu C.S., Liang Y. (2012). The effects of gold nanoparticles in wound healing with antioxidant epigallocatechin gallate and α-lipoic acid. J. Nanotechnol. Biol. Med..

[45] Jiang X., Sun D., Zhang G., He N., Liu H., Huang J., Odoom-Wubah T., Li Q. (2013). Investigation of active biomolecules involved in the nucleation and growth of gold nanoparticles by *Artocarpus heterophyllus* Lam leaf extract. J. Nanopart. Res..

[46] Pak Z.H., Abbaspour H., Karimi N., Fattahi A. (2016). Eco-friendly synthesis and antimicrobial activity of silver nanoparticles using *Dracocephalum moldavica* seed extract. Appl. Sci..

[47] Kasthuri J., Kathiravan K., Rajendiran N. (2009). Phyllanthin-assisted biosynthesis of silver and gold nanoparticles: A novel biological approach. J. Nanopart. Res..

[48] Elemike E.E., Onwudiwe D.C., Ekennia A.C., Ehiri R.C., Nnaji N.J. (2017). Phytosynthesis of silver nanoparticles using aqueous leaf extracts of *Lippia citriodora*: Antimicrobial, larvicidal and photocatalytic evaluations. Mater. Sci. Eng. C.

[49] Hasanzadeh M., Khalilzadeh B., Shadjou N., Karim-Nezhad G., Lotfali L., Kazeman I., Abnosi M.H. (2010). A new kinetic-mechanistic approach to elucidate formaldehyde electrooxidation on copper electrode. Electroanalysis.

[50] Xu Z.A., Gao N., Chen H.J., Dong S.J. (2005). Biopolymer and carbon nanotubes interface prepared by self-assembly for studying the electrochemistry of microperoxidase-11. Langmuir.

[51] Min K., Yoo Y.J. (2009). Amperometric detection of dopamine based on tyrosinase-SWNTs-Ppy composite electrode. Talanta.

[52] Bard A.J., Faulkner L.R. (2001). Electrochemical Methods: Fundamentals and Applications.

[53] Arotiba O.A., Baker P.G., Mamba B.B., Iwuoha E.I. (2011). The Application of electrodeposited poly(propyleneimine) dendrimer as an immobilisation layer in a simple electrochemical DNA biosensor. Int. J. Electrochem. Sci..

[54] Verma A., Saraf S.K. (2008). 4-Thiazolidinone—A biologically active scaffold. Eur. J. Med. Chem..

[55] Koca M., Servi S., Kirilmis C., Ahmedzade M., Kazaz C., Ozbek B., Otuk G. (2005). Synthesis and antimicrobial activity of some novel derivatives of benzofuran: Part 1. Synthesis and antimicrobial activity of (benzofuran-2-yl)(3-phenyl-3-methylcyclobutyl) ketoxime derivatives. Eur. J. Med. Chem..

[56] Dolman S.J., Gosselin F., Shea P.D., Davies I.W. (2006). Superior reactivity of thiosemicarbazides in the synthesis of 2-Amino-1,3,4-oxadiazoles. J. Org. Chem..

[57] Murphy S.T., Case H.L., Ellsworth E., Hagen S., Husband M., Jonnides T., Limberakis C., Marotti K.R., Ottolini A.M., Rauckhorst M. (2007). The synthesis and biological evaluation of novel series of nitrile-containing fluoroquinolones as antibacterial agents. Bioorg. Med. Chem. Lett..

[58] Espenti C.S., Krishna Rao K.S.V., Rao K.M. (2016). Bio-synthesis and characterization of silver nanoparticles using *Terminalia chebula* leaf extract and evaluation of its antimicrobial potential. Mater. Lett..

[59] Rao N.H., Lakshmidevi N., Pammi S.V.N., Kollu P., Ganapaty S., Lakshmi P. (2016). Green synthesis of silver nanoparticles using methanolic root extracts of *Diospyros paniculata* and their antimicrobial activities. Mater. Sci. Eng..

[60] Ajitha B., Reddy Y.A.K., Reddy P.S., Suneetha Y., Jeon H.-J., Ahn C.W. (2016). Instant biosynthesis of silver nanoparticles using *Lawsonia inermis* leaf extract: Innate catalytic, antimicrobial and antioxidant activities. J. Mol. Liq..

[61] Nayak D., Ashe S., Rauta P.R., Kumari M., Nayak B. (2016). Bark extract mediated green synthesis of silver nanoparticles: Evaluation of antimicrobial activity and antiproliferative response against osteosarcoma. Mater. Sci. Eng..

[62] Dharamaraj N., Viswanathamurthi P., Natarajan K. (2001). Ruthenium (II) complexes containing bidentate Schiff bases and their antifungal activity. Trans. Met. Chem..

[63] Okwu D.E., Okwu M.E. (2004). Chemical composition of *Spondias mombin* Linn. Plants parts. J. Sustain. Agric. Environ..

[64] Klein G., Kim J., Himmeldirk K., Cao Y., Chen X. (2007). Antidiabetes and anti-obesity activity of lagerstroemia speciosa. ECAM.

[65] Del-Rio A., Obdulio B.G., Castillo J., Marrin R.R., Ortuno A. (1997). Uses and properties of citrus flavonoids. J. Agric. Food Chem..

[66] Antolovich M., Prenzler P.D., Patsalides E., McDonald S., Robards K. (2002). Methods for testing antioxidant activity. Analyst.

[67] De-Leo M.E., Tranghee A., Passantino M., Mordente A., Lizzio M.M., Galeotti T., Zoli A. (2002). Manganese superoxide dismutase, glutathione peroxidase, and total radical trapping antioxidant capacity in active rheumatoid arthritis. J. Rheumatol..

[68] Prior L.R., Wu X., Schaich K. (2005). Standardized methods for the determination of antioxidant capacity and phenolics in foods and dietary supplements. J. Agric. Food Chem..

[69] Chandrasekaran R., Gnanasekar S., Seetharaman P., Keppanan R., Arockiaswamy W., Sivaperumal S. (2016). Formulation of *Carica papaya* latex-functionalized silver nanoparticles for its improved antibacterial and anticancer applications. J. Mol. Liq..

[70] Khan A.U., Wei Y., Ahmad A., Khan Z.U.H., Tahir K., Khan S.U., Muhammad N., Khan F.U., Yuan Q. (2016). Enzymatic browning reduction in white cabbage, potent antibacterial and antioxidant activities of biogenic silver nanoparticles. J. Mol. Liq..

[71] Swamy M.K., Akhtar M.S., Mohanty S.K., Sinniah U.R. (2015). Synthesis and characterization of silver nanoparticles using fruit extract of *Momordica cymbalaria* and assessment of their in vitro antimicrobial, antioxidant and cytotoxicity activities. Spectrochim. Acta.

[72] Kohsari I., Shariatinia Z., Pourmortazavi S.M. (2016). Antibacterial electrospun chitosan-polyethylene oxide nanocomposite mats containing bioactive silver nanoparticles. Carbohydr. Polym..

[73] Rajan R., Chandran K., Harper S.L., Yun S., Kalaichelvan P.T. (2015). Plant extract synthesized silver nanoparticles: An ongoing source of novel biocompatible materials. Ind. Crops Prod..

[74] Brands-Williams W., Cuvelier M.E., Berset C. (1995). Use of a free radical method to evaluate antioxidant activity. Lebens. Wiss. Technol..

